# Expanding the geography of evapotranspiration: An improved method to quantify land-to-air water fluxes in tropical and subtropical regions

**DOI:** 10.1371/journal.pone.0180055

**Published:** 2017-06-28

**Authors:** Daniela Jerszurki, Jorge L. M. Souza, Lucas C. R. Silva

**Affiliations:** 1 Wyler Department of Dryland Agriculture, French Associates Institute for Agriculture and Biotechnology of Drylands, Jacob Blaustein Institutes for Desert Research, Ben-Gurion University of the Negev, Sede Boqer, Israel; 2 Soil and Environment Studies Program, Federal University of Paraná, Curitiba, Paraná, Brazil; 3 Environmental Studies Program and Department of Geography, University of Oregon, Eugene, Oregon, United States of America; Pacific Northwest National Laboratory, UNITED STATES

## Abstract

The development of new reference evapotranspiration (*ETo*) methods hold significant promise for improving our quantitative understanding of climatic impacts on water loss from the land to the atmosphere. To address the challenge of estimating *ETo* in tropical and subtropical regions where direct measurements are scarce we tested a new method based on geographical patterns of extraterrestrial radiation (*Ra*) and atmospheric water potential (*Ψ*_*air*_). Our approach consisted of generating daily estimates of *ETo* across several climate zones in Brazil–as a model system–which we compared with standard *ETo*_*PM*_ (Penman-Monteith) estimates. In contrast with *ETo*_*PM*_, the simplified method (*ETo*_*MJS*_) relies solely on *Ψ*_*air*_ calculated from widely available air temperature (^o^C) and relative humidity (%) data, which combined with *Ra* data resulted in reliable estimates of equivalent evaporation (*E*_*e*_) and *ETo*. We used regression analyses of *Ψ*_*air*_ *vs ETo*_*PM*_ and *E*_*e*_ *vs ETo*_*PM*_ to calibrate the *ETo*_*MJS*(*Ψair*)_ and *ETo*_*MJS*_ estimates from 2004 to 2014 and between seasons and climatic zone. Finally, we evaluated the performance of the new method based on the coefficient of determination (*R*^*2*^) and correlation (*R*), index of agreement “*d*”, mean absolute error (*MAE*) and mean reason *(MR*). This evaluation confirmed the suitability of the *ETo*_*MJS*_ method for application in tropical and subtropical regions, where the climatic information needed for the standard *ETo*_*PM*_ calculation is absent.

## Introduction

The amount of water that flows through the soil-plant-atmosphere continuum is a key factor to be considered in ecosystem conservation and management efforts. Estimates of water fluxes from land-to-air are needed, for example, for the introduction of new crops, prediction of migration of plant species, and improvement of soil and irrigation management under climate change [1–3]. Assessing water fluxes in situ can be costly and time consuming and, depending on the method used, such assessments are subject to large uncertainties [4]. Baseline estimates of water fluxes are missing in many parts of the world, including the tropical and subtropical regions [5], owing to limited measurements of reference evapotranspiration (*ETo*).

Over the past 50 years, several methods have been developed to estimate the reference evapotranspiration [6–7]. The need to find a best model with minimum possible error relative to field measurements led to the Penman-Monteith model [6, 8–10], which is recognized as the standard method for agricultural regions worldwide [6, 9]. However, in areas that are dominated by natural ecosystems, especially those located in remote tropical and subtropical regions, the climate data needed for the application of the Penman-Monteith method are often unavailable [5]. Attempts to simplify the estimation of *ETo* using a small set of climatic variables, such air temperature and solar radiation, have been proposed [11–30]. The validation of simplified methods to estimate *ETo* has been mostly limited to climatic zones where they can be adjusted to fit Penman-Monteith projections, thus, overlooking vast tropical and subtropical regions [31–50].

In general, the literature that reports the performance of alternative methods against the standard Penman-Monteith method does so for specific climate conditions, missing the geographical variability of regional climates. This approach has proven inadequate for generating regional *ETo* estimates in countries that encompass multiple tropical and subtropical climatic conditions, such as Brazil [37–50]. The existing simplified *ETo* methods based on air temperature or on the combined effect of air temperature and solar radiation have shown either significant [39, 45] or not significant associations [48–49] to the Penman-Monteith method in tropical and subtropical climates. Those methods are thought to better match Penman-Monteith estimates in dry and warm climate zones [40, 50]. Under subtropical humid climates the methods based on solar radiation have shown the best adjustment to Penman-Monteith estimates [37, 41, 43]. Although those studies have contributed for the evaluation and choice of the most suitable *ETo* method within specific regions, they also show limitations for adequately estimating *ETo* across different climatic zones. Developing an alternative *ETo* method that is sensitive to regional climate heterogeneity in the tropics and sub-tropics is the central motivation of this study.

Among the most important climatic variables, vapor pressure deficit (*VPD*) exerts dominant influence on *ETo* estimations in different climate types [51–53]. However, for the coldest and wettest climates of tropical and subtropical regions in many parts of the world [51, 54–55], solar radiation also governs *ETo* variability. Thus, the use of solar radiation in combination to the *VPD* is a promising alternative to *ETo* estimates at scales that encompass multiple climatic regions. The solar radiation represents the total available latent energy to evapotranspiration process [56]. Among the existing radiation forms, the extraterrestrial radiation is easily estimated by use of latitude, hour of the day and solar constant (*G*_*cs*_). In the Penman-Monteith method, evaporative fluxes are mainly attributed to *VPD* which is related to the aerodynamic terms, such as wind speed [6]. Vapor pressure deficit in combination with latent heat drive soil water evaporation [57] and plant transpiration [4]. Thus, the resulting evapotranspiration is proportional to VPD and energy inputs [58] and the combined analysis of these variables allows for the study of the spatial heterogeneity of *ETo* [58].

Notably, the flux of water from soil and plants to the atmosphere is a result of the water potential gradients, with movement occurring toward the direction of the lowest water potential [57]. On average, the water vapor in the atmosphere represents the lowest state of energy (i.e., lowest water potential) along the soil-plant-atmosphere system. The study of water movement in the atmosphere can be complicated due variation in plant cover [4], species-specific water-use efficiency and transpiration rates [59], variability in water vapor pressure in relation to other gases, and climatic dynamism [60]. However, previous investigations of the water potential gradients along the soil-plant-atmosphere continuum have suggested that net water fluxes can be simplified to produce reliable *ETo* baselines that are important for management as well as conservation efforts aimed at mitigating the effects of climate change [57, 61–63]. Accordingly, here we propose an alternative *ETo* method based on atmospheric water potential and solar radiation, using a wide range of climate types in Brazil as a model system for improving tropical and subtropical land-to-air water flux estimates.

## Theoretical considerations

The basic principle that surrounds the notion of atmospheric water potential as a driving force of evapotranspiration, regardless of plant cover and soil properties, is rooted in the first and second laws of thermodynamics [57, 61]. Briefly, the balance of heat, mechanical work (*W*), and variation of internal energy (Δ*U*) of a system are considered to be in equilibrium at time zero:

Q−W−ΔU=0 (1)

where: *Q* is heat added to the system; *W* is the mechanical work; and, Δ*U* is the change in internal energy *U* of the system.

Considering changes in energy that trigger dynamic responses:

dU=dQ−dW (2)

where: *dU* is a differential function of *U*, depending only of initial and final state of a transformation; *dQ* is the differential of line function, representing the input and outputs of heat; and, *dW* is the differential of work, equal to *dQ* in adiabatic processes.

*dQ* is equal to *TdS*, where *S* is the entropy. The definition of *S* from the initial equilibrium (A) to the dynamic (B) state is given by:

$$S_{B}-S_{A}=\int_{A}^{B}\frac{dQ}{T}$$ (3)

Considering the second law of thermodynamics, which defines other energy functions of thermodynamic potential, the Gibbs free energy function is given by:

G=H−T⋅S=U+P⋅V−T⋅S (4)

The Gibbs free energy (*G*) is only dependent on the system state, i.e., the pressure (*P*), volume (*V*) and temperature (*T*). This function explains the available energy to make work, which is given by:

dG=V⋅dP−S⋅dT (5)

where: *G* is a function of *T* and *P*. Representing the Eq (5) in a mass (m) basis, considering *P = e*_*a*_ and *g* = *Ψ* in an isothermal path (*dT* = 0) and assuming the water vapor acting as an ideal gas we have:

$$d\psi =\frac{R\cdot T}{Mv}\cdot \frac{de_{a}}{e_{a}}$$ (6)

Integrating the equation from standard condition (*e*_*s*_) to actual condition (*e*_*a*_) we have:

$$\Delta \psi_{air}=\int_{e_{s}}^{e_{a}}\frac{R\cdot T}{Mv}\cdot \frac{de_{a}}{e_{a}}=\frac{R\cdot T}{Mv}\cdot \ln\left(\frac{e_{a}}{e_{s}}\right)$$ (7)

Due to the difficult of measurement of the absolute *Ψ*_*air*_, between the standard (*Ψ*_*o*_) and interest condition (*Ψ*_*air*_), we set *Ψ*_*o*_ = 0 and Δ*Ψ*_*air*_ = *Ψ*_*air*_:

$$\psi_{air}=\frac{R\cdot T}{Mv}\ln\left(\frac{e_{a}}{e_{s}}\right)$$ (8)

where: *Ψ*_*ai*r_ is the atmospheric water potential (MPa); *R* is the gas constant (8.314 J mol^−1^ K^−1^); *T* is the absolute temperature (K); *e*_*a*_ is the actual vapor pressure (MPa); *e*_*s*_ is the saturated vapor pressure (MPa); and, *Mv* is the partial molar volume of water (18.10^−6^ m^3^ mol^–1^).

In order to combine the effect of *Ψ*_*air*_ to extraterrestrial radiation (*Ra*) in the equivalent water evaporation, the *Ψ*_*air*_ is turned into a coefficient of proportionality *K*_*Ψair*_, ranging from 0 to 1:

$$K_{\psi air}=\left|\frac{\psi_{air.i}-\psi_{air.\min}}{\psi_{air.\max}-\psi_{air.\min}}\right|$$ (9)

where: *K*_*Ψair*_ is the coefficient of proportionality of *Ψ*_*air*_ (dimensionless); *Ψ*_*ai*r.*i*_ is the atmospheric water potential at the *i*-day (MPa); *Ψ*_*air*.*max*_ is the maximum atmospheric water potential at the analyzed period (MPa); *Ψ*_*air*.*min*_ is the minimum atmospheric water potential at the analyzed period (MPa).

The equivalent water evaporation (*E*_*e*_−mm d^–1^) is obtained by transformation of *Ra* (MJ m^–2^ d^–1^) by use of the inverse constant of the latent heat of vaporization (1/λ) [6] multiplied by *K*_*Ψair*_.

$$Ra=\frac{24\cdot \left(60\right)}{\pi }\cdot G_{sc}\cdot dr\cdot \left[\omega_{s}\cdot sen\;\left(\varphi \right)\cdot sen\;\left(\delta \right)+\cos\;\left(\varphi \right)\cdot \cos\;\left(\delta \right)\cdot sen\;\left(\omega_{s}\right)\right]$$ (10)

$$E_{e}=K_{\psi air}\cdot \frac{R_{a}}{\lambda }$$ (11)

where: *Ra* is the extraterrestrial radiation (MJ m^–2^ d^–1^); *G*_*sc*_ is the solar constant (*G*_*sc*_ = 0.0820 MJ m^-2^ min^-1^); *dr* is the relative distance Earth–Sun (dimensionless); *ω*_*s*_ is the hourly angle corresponding to sunset (rad); *φ* is the latitude (rad); *δ* is the inclination of the sun (rad); *E*_*e*_ is the equivalent water evaporation obtained by solar radiation and weighted by atmospheric water potential at each *i-*day (mm d^–1^); *K*_*Ψair*_ is the coefficient of proportionality of atmospheric water potential (dimensionless); *λ* is the latent heat of vaporization (*λ* = 2.45 MJ kg^–1^). The estimated *E*_*e*_ can then be converted into *ETo* as explained in the calibration step described below.

## Material and methods

### Climate data

To perform the calculations described above in Brazil as a case study we used a set of nine meteorological stations [64] (Table 1) distributed across the most representative climatic zones of that country (Table 2) [65]. We relied on daily observations of maximum, minimum, and average air temperature (^o^C), relative humidity (%), daily sunshine hours (MJ m^-2^ d^-1^), and wind speed (m s^–1^) measured at ten meters above the ground level, from January 2004 to January 2014. Daily sunshine hours were measured by the heliograph Campbell-Stokes (model 240-1070-L) at hourly intervals. Daily wind speed was obtained by the anemometer Vaisala WT521. Wind speed measurements were transformed to wind speed at 2 m height by the wind profile relationship [6]. Daily air temperature and relative humidity were obtained by the thermometer Fluke 5699 and the humidity sensor Vaisala HMK15, respectively.

**Table 1:** Climate classification, location and coordinates of the Brazilian meteorological stations used in this study.

Climate	State	Station	Latitude (degree S)	Longitude (degree W)	Altitude (m)
Af	Amazonas	Manaus	–3.10	–60.01	61.25
Am	Amapá	Macapá	–0.05	–51.11	14.46
As	Sergipe	Aracaju	–10.95	–37.01	4.72
Aw	Goiás	Goiânia	–16.66	–49.25	741.48
Bsh	Pernambuco	Petrolina	–9.38	–40.48	370.46
Cfa	Rio Grande do Sul	Porto Alegre	–30.05	–51.16	46.97
Cfb	Paraná	Curitiba	–25.43	–49.26	923.50
Cwa	Minas Gerais	Uberaba	–19.73	–47.95	737.00
Cwb	Minas Gerais	Belo Horizonte	–19.93	–43.93	915.00

**Table 2:** Koeppen’s climate classification based on temperature and precipitation at each location.

Symbol	Temperature (^o^C)			Rainfall (mm)			Climate
				Monthly		Annual	
	T_1_	T_2_	T_3_	R_d_	R_w_		
Af	≥ 18			≥60		≥25(100–R_d_)	Tropical without dry season
Am					< 60		Tropical monsoon
As						<25(100–R_sdry_)	Tropical with dry summer
Aw						<25(100–R_wdry_)	Tropical with dry winter
Bsh			≥18			<5.R_LIM_	Semi-arid with low latitude and altitude
Cfa	–3<T<18	≥22		>40			Humid subtropical, oceanic climate without dry season and with hot summer
Cfb		4≤T_M10<_22					Humid subtropical, oceanic climate without dry season, with temperate summer
Cwa	–3<T<18	≥22		<40			Humid subtropical with dry winter and hot summer
Cwb		4≥T_M10<_22			R_swet_≥10.R_wwet_		Humid subtropical with dry winter and temperate summer

T1 –temperature of the coldest month;T2 –temperature of the hottest month; T3 –annual mean temperature; Rd−rainfall of the driest month; Rw−rainfall of the wettest month; R_sdry_−rainfall of the driest month on summer; Rwdry−rainfall of the driest month on winter; R_swet_−rainfall of the wettest month on summer; R_wwet_−rainfall of the wettest month on winter; R_LIM_−rainfall of the driest month of the year; T_M10_ –number of months where the temperature is above 10^°^C.

### Penman-Monteith reference evapotranspiration (*ETo*_*PM*_)

The Penman-Monteith method estimates *ETo* as follows [9]:

$$ETo_{PM}=\frac{0,408\cdot \Delta \cdot \left(R_{n}-G\right)+\gamma_{psy}\cdot \frac{C_{n}}{\left(T_{air}+273\right)}\cdot u_{2}\cdot \left(e_{s}-e_{a}\right)}{\Delta +\gamma_{psy}\cdot \left(1+C_{d}\cdot u_{2}\right)}$$ (12)

where: *ETo*_*PM*_*−*reference evapotranspiration (mm d^–1^); Δ–slope of the saturated water-vapor-pressure curve (kPa ^o^C^–1^); *R*_*n*_−net radiation at the crop surface (MJ m^–2^ d^–1^); *G*–soil heat flux (MJ m^–2^ d^–1^); *γ*_psy_−psychrometric constant (kPa ^o^C^–1^); *T*_*air*_−average daily air temperature (^o^C); *u*_*2*_ –wind speed at two meters height (m s^–1^); *e*_*s*_−saturated vapor pressure (kPa); *e*_*a*_−actual vapor pressure (kPa); *C*_*n*_−constant related to the reference type and calculation time step, considered equal to 900 for grass (dimensionless); *C*_*d*_−constant related to the reference type and calculation time step, considered equal to 0.34 for grass (dimensionless).

Daily vapor pressure deficit (*e*_*s*_*−e*_*a*_) is estimated by the difference between saturated and actual vapor pressure. Saturated vapor pressure is calculated using air temperature based on the Tetens formula [66]. Actual vapor pressure is obtained by saturated vapor pressure multiplied by fractional humidity. Daily net radiation (*Rn*) is estimated by the difference between net longwave and shortwave radiation. The net longwave radiation (*Rnl*) is obtained by relative shortwave radiation (*Rs*/*Rso*), air temperature and actual vapor pressure. The net shortwave radiation (*Rns*) is obtained from solar radiation (*Rs*) measurements, which are determined by the relation between extraterrestrial radiation (*Ra*) and relative sunshine duration (*n*/*N*) [6]. Finally, soil heat flux (*G*) is calculated using air temperature [67].

### Alternative “Moretti-Jerszurki-Silva” method: *ETo*_*MJS(Ψair)*_ and *ETo*_*MJS*_

The alternative “Moretti-Jerszurki-Silva” method is easily calibrated and used to estimate the *ETo*. The method is proposed based on *Ψ*_*air*_ (*ETo*_*MJS(Ψair)*_); and, on *Ψ*_*air*_ and *Ra* by estimation of *E*_*e*_ (*ETo*_*MJS*_). Daily values of *Ψ*_*air*_ (Eq 8) *vs ETo*_*PM*_ and *E*_*e*_ (Eq 11) *vs ETo*_*PM*_ obtained from meteorological stations are adjusted from regression analysis in a monthly and annual basis, between 2004 and 2011. As a general practice in validation procedures, an independent dataset should be used to fit the model; accordingly, the performance assessment and validation of *ETo*_*MJS(Ψair)*_ and *ETo*_*MJS*_ against *ETo*_*PM*_ are determined based on regression analysis for the last two years of the time series (January of 2012 –January of 2014). *ETo*_*MJS(Ψair)*_ and *ETo*_*MJS*_ values are obtained using coefficients "*a*" and "*b*" for *Ψ*_*air*_ *vs ETo*_*PM*_ and *E*_*e*_ *vs ETo*_*PM*_, respectively, between 2004 and 2011.

$$ETo_{MJ(\psi air)\;i}=a+b.\;\psi_{air\;i}$$ (13)

$$ETo_{MJ\;i}=a+b.\;E_{e}$$ (14)

where: *ETo*_*MJS(Ψair)*.*i*_ is the calibrated reference evapotranspiration estimated by atmospheric water potential at each *i-*day (mm d^–1^); *Ψ*_*air*.*i*_ is the atmospheric water potential at each *i*-day (MPa); *ETo*_*MJS*.*i*_ is the calibrated reference evapotranspiration estimated by atmospheric water potential and solar radiation at each *i-*day (mm d^–1^); *E*_*e*_ is the equivalent evaporation obtained by solar radiation and weighted by atmospheric water potential at each *i-*day (mm d^–1^); *a* is the linear coefficient (mm d^–1^); *b* is the angular coefficient (dimensionless).

### Validation of *ETo*_*MJS(Ψair)*_ and *ETo*_*MJS*_ estimates using lysimetric measurements

In addition to the 10-years comparison with standard *ETo*_*PM*_ in multiple climatic zones, described above, a seasonal validation of the new *ETo*_*MJS(Ψair)*_ and *ETo*_*MJS*_ method was conducted in situ using available lysimetric measurements (*ETo*_*LIS*_) located at a reference pasture plantation. This independent validation was performed at a site of typical semi-arid climate type *Bsh* (latitude 3^o^18’S, longitude 39^o^12’W at an altitude of 30 m above the sea level) using climatic data collected between 1997 and 1998 as well *ETo*_*LIS*_ previously reported in the literature [68]. The validation could only be performed at this site and period due to the scarcity of co-located *ETo*_*LIS*_ and reliable weather stations in other climatic regions. This is sufficient, however, to demonstrate that the proposed method holds in the analysis of both seasonal and multi-year *ET* patterns without the need for the detailed climatic data that is required for the standard *ETo*_*PM*_ calculation. As described above, the new *ETo*_*MJS(Ψair)*_ (Eqs 8 and 13) and *ETo*_*MJS*_ (Eqs 8–11 and 14) were estimated using only air temperature, relative humidity and altitude. The calibration of *ETo*_*MJS(Ψair)*_ (Eq 13) and *ETo*_*MJS*_ (Eq 14) were carried out using the monthly coefficients "*a*" and "*b*" of the linear relation between *Ψ*_*air*_ *vs ETo*_*PM*_ (March: *a* = 2.39 mm d^–1^ and *b* = –0.043; April: *a* = 2.59 mm d^–1^ and *b* = –0.036; May: *a* = 2.20 mm d^–1^ and *b* = –0.035; and, June: *a* = 2.09 mm d^–1^ and *b* = –0.033), and *E*_*e*_ *vs ETo*_*PM*_ (March: *a* = 2.74 mm d^–1^ and *b* = 0.47; April: *a* = 2.84 mm d^–1^ and *b* = 0.44; May: *a* = 2.44 mm d^–1^ and *b* = 0.48; and, June: *a* = 2.34 mm d^–1^ and *b* = 0.47), obtained in the present study for the semi-arid climate subgroup, between 2004–2011. Finally, *ETo*_*MJS(Ψair)*_ and *ETo*_*MJS*_ were regressed against *ETo*_*LIS*_.

### Statistics

We used coefficients of variation (CV) to assess the variability of *ETo*_*PM*_ in response to climatic data collected across sites between 2004 and 2014. We relied on multiple regression analyses to correlate the estimated *ETo*_*PM*_ to climatic variables for each specific climatic zone (Table 1). We then compared daily reference evapotranspiration obtained with the alternative method to standard daily *ETo*_*PM*_ and/or *ETo*_*LIS*_ (validation) using regression analysis. The goodness of fit of the alternative methods was obtained by use of *R*^*2*^ and *R* as an index of precision and correlation, and agreement index “*d*” as an index of accuracy [69]. The agreement index is a measure of the effectiveness with which the alternative method estimates the Penman-Monteith reference evapotranspiration, considering the dispersion of the data relative to the 1:1 line:

$$d=1-[\frac{\sum_{i=1}^{n}(ETo_{alternativei}-ETo_{i}{)}^{2}}{\sum_{i=1}^{n}\;{\left(\left|ETo_{alternativei}-\bar{ETo}\right|+\left|ETo_{i}-\bar{ETo}\right|\right)}^{2}}]$$ (15)

where: *d* is the agreement index (dimensionless); *ETo*_*alternative*.*i*_ is the reference evapotranspiration estimated by alternative method at each *i-*day (mm d^–1^); *ETo*._*i*_ is the reference evapotranspiration estimated by Penman-Monteith method or measured in the lysimeters at each *i-*day (mm d^–1^); $\bar{ETo}$ is the average reference evapotranspiration estimated by Penman-Monteith method or measured in the lysimeters (mm d^–1^).

For further comparison, the mean absolute error (*MAE*) and the mean ratio (*MR*) [70] were used to evaluate the reference evapotranspiration estimated by atmospheric water potential:

$$MAE=\frac{1}{n}\cdot \sum_{i=1}^{n}\left(\left|ETo_{alternativei}-ETo_{i}\right|\right)$$ (16)

$$MR=\frac{1}{n}\sum_{i=1}^{n}\frac{ETo_{alternativei}}{ETo_{i}}$$ (17)

where: *ETo*_*alternative*.*i*_ is the reference evapotranspiration estimated by the alternative method at each *i-*day (mm d^–1^); *ETo*_*i*_ is the reference evapotranspiration estimated by Penman-Monteith method or measured in the lysimeters at each *i-*day (mm d^–1^); *n* is the number of observations (dimensionless). Finally, *MAE* was used to measure the accuracy of the proposed method and *MR* was used as an index of under- or overestimation of the standard *ETo*_*PM*_, such that when standard and alternative data are similar, *MAE* is close to zero and *MR* is close to one, indicating a more accurate estimation.

## Results

As expected, our observations showed large variability of all climatic parameters (*T*_*max*_, *T*_*min*_, *RH*, *Rs*, *u*_*2*_ and *VPD*) across the different climate zones sampled throughout Brazil. The results described here span *ETo* trends in humid subtropical, tropical with dry summers, and semi-arid regions (Table 3). In general, *VPD* was the most seasonally variable parameter in humid climatic zones, reaching its lowest values during wet summers. Across sites, high *T*_*max*_ and *T*_*min*_ and low *RH* in semi-arid climate resulted in the highest *VPD* and *ETo*_*PM*_. These results reflect the geographical influence–governed by variation in atmospheric water potential and *Rs–*on *ETo* throughout the country.

**Table 3:** Annual daily average, coefficient of variation of climatic variables and coefficient of correlation between *ETo*_*PM*_ and climatic variables between 2004 and 2014 at different climatic zones.

Clim	Variable	Aver	CV (%)					R (dimensionless)				
			A	Sum	Aut	Win	Spr	A	Sum	Aut	Win	Spr
Af	*T*_*min*_ (^o^C)	22.74	2.64	1.34	1.54	1.61	1.50	0.2	0.0	0.6	0.1	-0.1
	*T*_*max*_ (^o^C)	29.83	2.42	1.33	1.75	1.97	1.45	0.7	0.2	0.8	0.7	0.2
	*RH* (%)	73.77	3.14	1.89	1.88	2.52	2.05	-0.5	-0.2	-0.3	-0.8	-0.5
	*VPD* (kPa)	0.66	13.00	4.71	6.59	10.80	7.40	0.8	0.7	0.7	0.9	0.7
	*Rs* (MJ m^–2^ d^–1^)	18.06	9.54	3.17	7.53	8.51	3.47	1.0	0.9	1.0	1.0	0.9
	*u*_*2*_ (m s^–1^)	1.73	8.45	6.00	6.11	7.00	6.79	0.2	0.3	-0.3	0.3	0.5
	*ETo*_*PM*_ (mm d^–1^)	3.73	11.10	3.37	8.67	9.97	3.22	─	─	─	─	─
Am	*T*_*min*_ (^o^C)	22.22	4.03	0.48	2.94	1.71	1.63	0.5	-0.1	1.0	0.8	0.5
	*T*_*max*_ (^o^C)	30.37	2.31	0.49	2.06	1.31	0.80	0.6	-0.1	1.0	0.9	0.6
	*RH* (%)	79.02	2.30	0.49	0.98	2.09	1.05	-0.5	-0.6	-0.9	-1.0	0.0
	*VPD* (kPa)	0.76	10.96	2.34	8.02	9.93	2.81	0.6	0.5	1.0	1.0	0.9
	*Rs* (MJ m^–2^ d^–1^)	18.56	10.13	2.53	6.97	8.05	2.08	0.6	0.9	1.0	1.0	0.9
	*u*_*2*_ (m s^–1^)	1.98	13.84	3.89	7.19	11.90	2.86	0.6	0.7	0.9	1.0	0.6
	*ETo*_*PM*_ (mm d^–1^)	3.97	15.71	14.24	9.84	10.74	2.27	─	─	─	─	─
As	*T*_*min*_ (^o^C)	23.41	4.52	0.87	2.26	1.83	2.03	0.7	0.7	0.9	0.6	0.3
	*T*_*max*_ (^o^C)	30.01	2.14	0.33	1.44	0.93	0.86	0.7	0.6	0.9	0.9	0.3
	*RH* (%)	78.41	3.95	1.71	1.07	3.02	0.85	-0.9	-0.9	-0.8	-1.0	-0.7
	*VPD* (kPa)	0.78	16.46	6.67	7.21	11.90	2.57	1.0	0.9	0.9	1.0	0.8
	*Rs* (MJ m^–2^ d^–1^)	21.04	11.49	3.76	5.55	10.29	2.32	1.0	0.9	0.9	1.0	0.9
	*u*_*2*_ (m s^–1^)	3.14	14.53	8.26	7.07	8.90	5.50	0.7	0.8	-0.7	0.9	0.4
	*ETo*_*PM*_ (mm d^–1^)	4.45	14.12	4.46	6.85	12.33	2.40	─	─	─	─	─
Aw	*T*_*min*_ (^o^C)	20.68	7.42	0.52	5.76	6.13	1.27	0.7	0.7	0.1	0.7	-0.2
	*T*_*max*_ (^o^C)	31.45	3.23	0.70	1.22	3.66	2.06	0.8	0.7	0.5	0.8	0.3
	*RH* (%)	71.74	11.27	1.67	4.76	6.84	6.80	-0.9	-0.8	-0.6	-0.9	-0.7
	*VPD* (kPa)	0.98	31.28	8.09	12.58	16.88	17.82	0.9	0.8	0.6	0.9	0.7
	*Rs* (MJ m^–2^ d^–1^)	18.98	6.87	3.02	3.09	6.23	3.38	1.0	0.9	0.9	1.0	0.9
	*u*_*2*_ (m s^–1^)	1.47	12.39	6.61	7.39	8.57	5.45	0.9	0.7	0.4	0.9	0.0
	*ETo*_*PM*_ (mm d^–1^)	4.09	12.83	4.17	4.02	12.49	5.68	─	─	─	─	─
Bsh	*T*_*min*_ (^o^C)	22.01	5.86	0.97	3.74	2.32	2.63	0.5	0.3	0.8	0.5	0.4
	*T*_*max*_ (^o^C)	32.14	4.66	1.22	2.50	3.58	1.29	0.9	0.7	0.9	0.9	0.6
	*RH* (%)	55.37	10.49	5.20	2.73	8.18	6.55	-0.8	-0.8	0.4	-0.9	-0.2
	*VPD* (kPa)	1.59	18.44	11.93	6.26	15.94	4.78	0.9	0.8	0.4	0.9	0.5
	*Rs* (MJ m^–2^ d^–1^)	17.71	11.98	4.13	8.55	10.75	3.19	0.9	0.7	0.9	0.9	0.7
	*u*_*2*_ (m s^–1^)	2.28	12.34	8.36	10.05	6.23	6.82	0.0	0.8	-0.5	0.4	0.2
	*ETo*_*PM*_ (mm d^–1^)	4.28	16.12	8.21	7.50	15.00	3.51	─	─	─	─	─
Cfa	*T*_*min*_ (^o^C)	16.44	20.84	2.11	15.70	8.00	9.06	0.9	0.1	1.0	0.8	0.9
	*T*_*max*_ (^o^C)	24.93	13.94	1.88	10.23	4.32	7.57	0.9	0.5	1.0	0.8	1.0
	*RH* (%)	78.51	3.50	2.16	2.40	2.26	2.51	-0.9	-0.8	-0.9	-0.8	-0.9
	*VPD* (kPa)	0.57	30.30	7.98	22.72	13.88	17.42	1.0	0.9	1.0	0.8	1.0
	*Rs* (MJ m^–2^ d^–1^)	17.01	26.51	8.14	17.88	18.17	9.80	1.0	1.0	1.0	1.0	1.0
	*u*_*2*_ (m s^–1^)	2.57	19.84	9.01	14.01	16.28	6.30	0.8	0.7	0.9	0.9	0.2
	*ETo*_*PM*_ (mm d^–1^)	3.14	37.78	8.91	27.66	26.88	14.01	─	─	─	─	─
Cfb	*T*_*min*_ (^o^C)	13.08	2.57	2.57	2.57	2.57	2.57	0.9	0.2	1.0	0.7	0.9
	*T*_*max*_ (^o^C)	23.43	23.53	2.57	19.05	10.74	9.92	0.9	0.3	1.0	0.8	0.9
	*RH* (%)	81.22	11.33	2.03	8.88	5.09	7.14	-0.2	-0.5	-0.1	-0.5	-0.8
	*VPD* (kPa)	0.44	2.46	1.35	1.26	2.91	2.27	0.9	0.5	0.9	0.8	0.9
	*Rs* (MJ m^–2^ d^–1^)	16.31	19.44	7.23	14.55	16.53	15.99	1.0	1.0	1.0	1.0	1.0
	*u*_*2*_ (m s^–1^)	2.10	20.53	6.66	14.17	13.60	8.99	0.7	0.5	0.5	0.6	0.5
	*ETo*_*PM*_ (mm d^–1^)	2.78	12.64	7.50	8.56	9.49	6.36	─	─	─	─	─
Cwa	*T*_*min*_ (^o^C)	17.12	16.96	4.00	15.24	14.32	5.75	0.5	-0.2	0.7	0.9	-0.3
	*T*_*max*_ (^o^C)	29.95	6.28	4.08	5.74	7.66	4.73	0.7	0.5	0.7	0.9	0.4
	*RH* (%)	65.88	15.21	4.37	7.30	13.39	11.79	-0.3	-0.7	0.3	-0.7	-0.7
	*VPD* (kPa)	1.06	30.15	14.98	12.74	23.38	26.75	0.5	0.7	0.3	0.9	0.7
	*Rs* (MJ m^–2^ d^–1^)	18.69	13.04	9.63	11.14	11.36	11.71	0.9	0.8	0.9	1.0	0.8
	*u*_*2*_ (m s^–1^)	1.11	41.10	31.42	36.95	31.37	33.95	0.4	0.1	0.3	0.9	0.4
	*ETo*_*PM*_ (mm d^–1^)	3.86	19.91	12.06	15.92	20.90	13.09	─	─	─	─	─
Cwb	*T*_*min*_ (^o^C)	17.38	11.30	1.44	10.14	6.86	3.35	0.9	0.6	1.0	0.9	0.2
	*T*_*max*_ (^o^C)	27.21	5.20	1.98	4.79	4.30	1.96	0.9	0.5	1.0	0.9	0.6
	*RH* (%)	68.10	7.52	3.31	2.96	4.98	6.60	0.3	-0.6	0.7	-0.7	-0.2
	*VPD* (kPa)	0.92	13.67	10.09	5.78	13.62	14.77	0.3	0.6	0.7	0.9	0.4
	*Rs* (MJ m^–2^ d^–1^)	18.52	12.58	5.78	9.38	9.65	5.34	1.0	1.0	1.0	1.0	0.9
	*u*_*2*_ (m s^–1^)	1.50	10.37	9.04	6.41	11.05	8.96	0.5	0.7	0.4	0.9	0.1
	*ETo*_*PM*_ (mm d^–1^)	3.61	18.81	5.94	15.10	17.60	5.23	─	─	─	─	─

Clim–climate type; Aver–annual daily average; A–annual; Sum–summer; Aut–autumn; Win–winter; Spr–spring.

### Adjustment of atmospheric water potential and performance of the alternative methods *ETo*_*MJS(Ψair)*_ *and ETo*_*MJS*_

**Fig 1:**
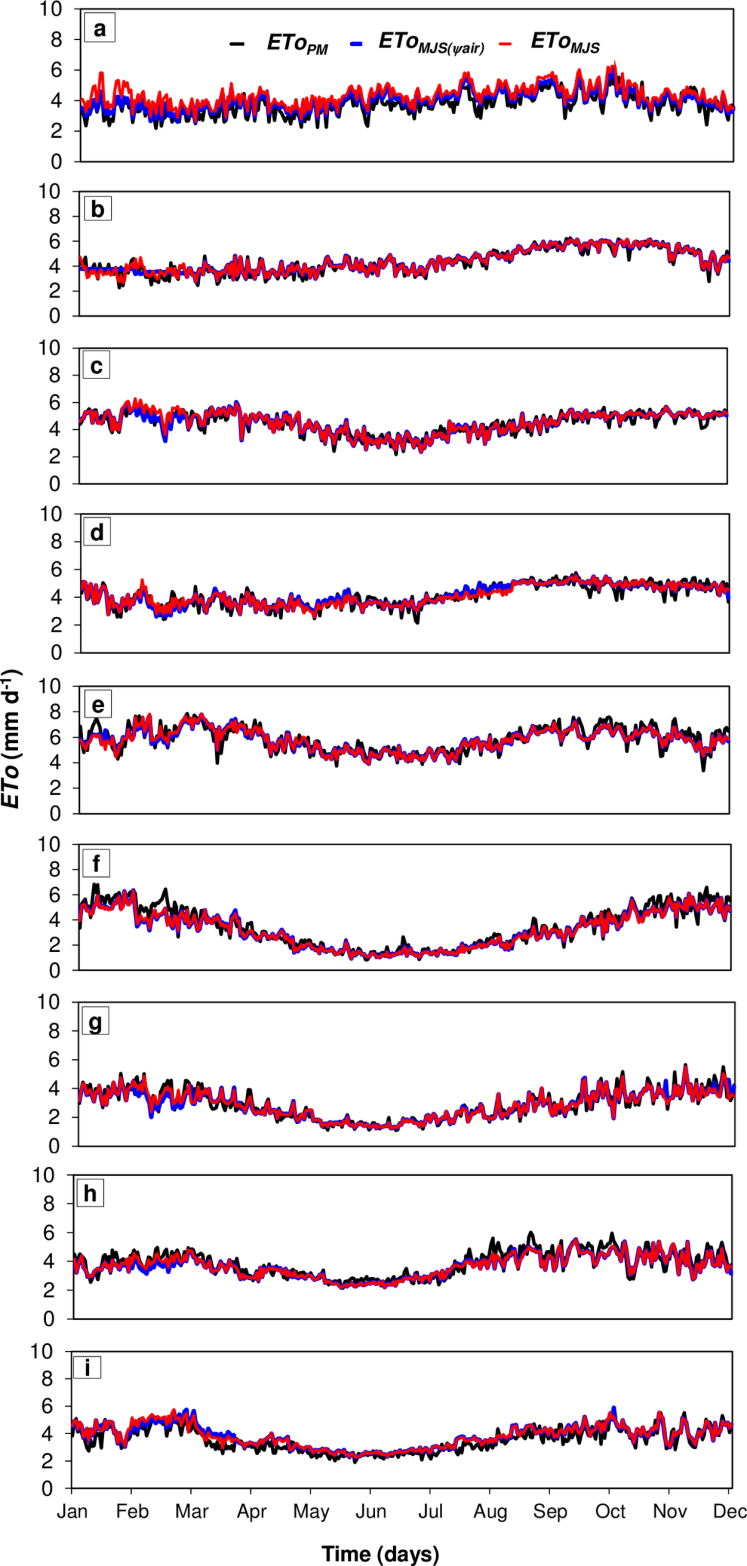
Daily reference evapotranspiration estimated by *ETo*_*MJS(*_*Ψ*_*air)*_ and *ETo*_*MJS*_ alternative methods, between 2012 and 2014, for the climate types: (a) Af; (b) Am; (c) As; (d) Aw; (e) Bsh; (f) Cfa; (g) Cfb; (h) Cwa; and, (i) Cwb.

We identified a strong negative linear relationship between *Ψ*_*air*_ and *ETo*_*PM*_ (P<0.05), with the coefficients "*a*" and "*b*" varying with climatic zone (S1 Fig). The linear coefficient “*a*” corresponds to other climatic variables that in addition to *Ψ*_*air*_ drive atmospheric water demand (*VPD*) and control *ETo*_*PM*_, while coefficient “*b*” corresponds to the rate of change in the *ETo*_*PM*_ relative to *Ψ*_*air*_. Even though *Ψ*_*air*_ is not used in the calculation of the standard *ETo*_*PM*_ (Eq 12), it strongly affects its variability over time and space. The exception occurred at the humid subtropical site, which showed the smallest linear coefficients between *Ψ*_*air*_ and *ETo*_*PM*_, due to lower magnitudes of *ETo*_*PM*_. We also identified an *ETo* threshold (2 mm d^–1^) beyond which evapotranspiration is primarily controlled by solar radiation and wind speed (S1 Fig). The highest angular coefficients |"*b*"| were observed in the tropical climate with dry winter (Aw) and the lowest in the subtropical climate. Accordingly, stronger associations and lower errors of adjustment in *ETo*_*MJS(Ψair)*_ were observed for tropical and semi-arid climates (S2 Fig and Table 4). Across all climate zones, the lowest associations between *ETo*_*MJS(Ψair)*_ and *ETo*_*PM*_ were observed during dry winter months (Fig 1).

**Table 4:** Performance of *ETo*_*MJS(Ψair)*_ and *ETo*_*MJS*_ as assessed based on *R* (coefficient of correlation), *d* (agreement index), *MAE* (mean absolute error), and *MR* (mean ratio) for all climate zones in an annual basis, between 2012 and 2014.

Climate	R		“d” Index		MAE		MR	
	(dimensionless)				(mm d^–1^)		(dimensionless)	
	*ETo*_*MJS(Ψair)*_	*ETo*_*MJS*_	*ETo*_*MJS(Ψair)*_	*ETo*_*MJS*_	*ETo*_*MJS(Ψair)*_	*ETo*_*MJS*_	*ETo*_*MJS(Ψair)*_	*ETo*_*MJS*_
Af	0.82	0.84	0.88	0.87	0.45	0.47	1.10	1.11
Am	0.91	0.91	0.95	0.95	0.34	0.33	1.03	1.03
As	0.82	0.87	0.90	0.93	0.43	0.35	1.03	1.02
Aw	0.84	0.84	0.91	0.92	0.40	0.40	1.03	1.03
Bsh	0.81	0.88	0.88	0.92	0.58	0.46	0.99	0.99
Cfa	0.71	0.89	0.77	0.94	1.07	0.62	1.16	1.06
Cfb	0.61	0.84	0.69	0.90	0.77	0.51	1.09	1.04
Cwa	0.58	0.75	0.67	0.82	0.79	0.64	1.02	0.99
Cwb	0.48	0.77	0.57	0.84	0.70	0.57	1.11	1.11

We also identified a linear relationship (P<0.05) between *E*_*e*_ and *ETo*_*PM*_ (S3 Fig), which demonstrates the suitability of using *Ψ*_*air*_ and *Ra*–sole drivers of *E*_*e*_−as the key parameters in the alternative method. The linear coefficients "*a*" were around: 3 mm day^–1^ for the semi-arid climate; 1.5 to 2.5 mm day^–1^ for tropical climates; and 1.0 to 2.5 mm day^–1^ for subtropical climates. The angular coefficients | "*b*" | ranged from 0.35 to 0.5 for subtropical climates; 0.4 for the semi-arid climate; and between 0.27 and 0.33 for tropical climates (S3 Fig). The calibration process, which involved the relation between *E*_*e*_ and *ETo*_*PM*_, further improved the performance of the alternative method *ETo*_*MJS*_ for subtropical climates (Fig 1, S4 Fig and Table 4).

The smallest adjustment errors (MAE and MR) of *ETo*_*MJS(Ψair)*_ and *ETo*_*MJS*_ were observed in the tropical climate types (Table 4). The smallest associations between *ETo*_*MJS*_ *vs ETo*_*PM*_ were observed in winter months, which is to be expected given the relatively low *Ra* and *ETo*_*PM*_ typical of this season [71].

**Fig 2:**
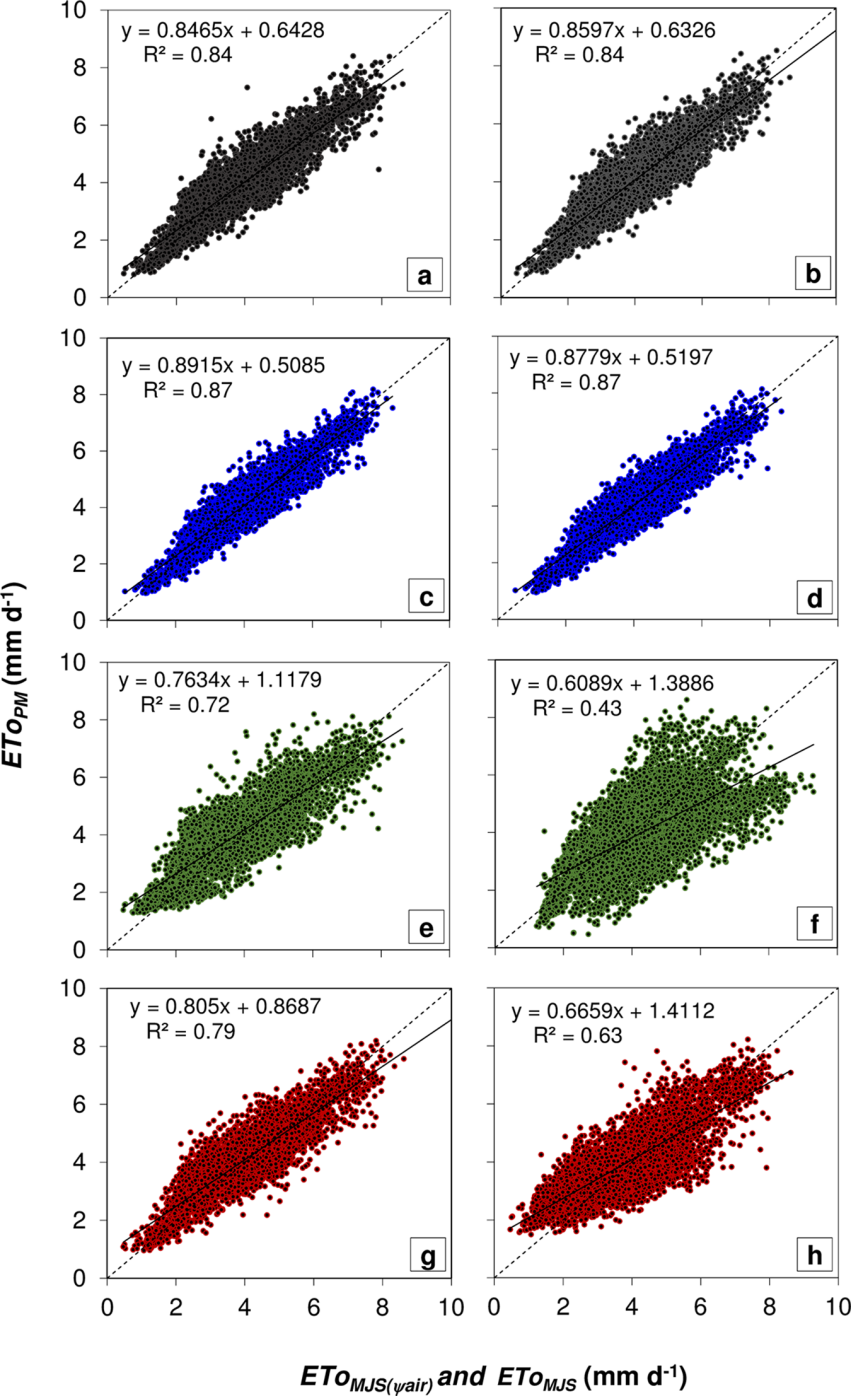
Daily reference evapotranspiration estimated by Penman-Monteith method as a response of reference evapotranspiration estimated between 2012 and 2014, considering different monthly “*a*” and “*b*” coefficients for each climate type for (a) *ETo*_*MJS(Ψair)*_ and (b) *ETo*_*MJS*_; different monthly average “*a*” and “*b*” coefficients for each climate subgroup for (c) *ETo*_*MJS(Ψair)*_ and (d) *ETo*_*MJS*_; different seasonal average “*a*” and “*b*” coefficients for each climate subgroup for (e) *ETo*_*MJS(Ψair)*_ and (f) *ETo*_*MJS*_; and, different annual average “*a*” and “*b*” coefficients for each climate subgroup for (g) *ETo*_*MJS(Ψair)*_ and (h) *ETo*_*MJS*_.

In an attempt to establish generic "*a*" and "*b*" coefficients for the different climate zones and seasons, we used the linear and angular coefficients determined at the three main climate groups: tropical, semi-arid and sub-tropical. This resulted in a predictable ET trend response to *Ψ*_*air*_ and *E*_*e*_. The highest significant *ETo*_*MJS(Ψair)*_ and *ETo*_*MJS*_ associations with *ETo*_*PM*_ were observed when using monthly average coefficients for the climate subgroups: humid tropical (Af, Am, As and Aw), semi-arid (*Bsh*), humid subtropical without dry season (Cfa and Cfb) and humid subtropical climates with dry summers (Cwa and Cwb) (Fig 2).

### Validation of the alternative method “Moretti-Jerszurki-Silva”: *ETo*_*MJS(Ψair)*_ and *ETo*_*MJS*_

**Fig 3:**
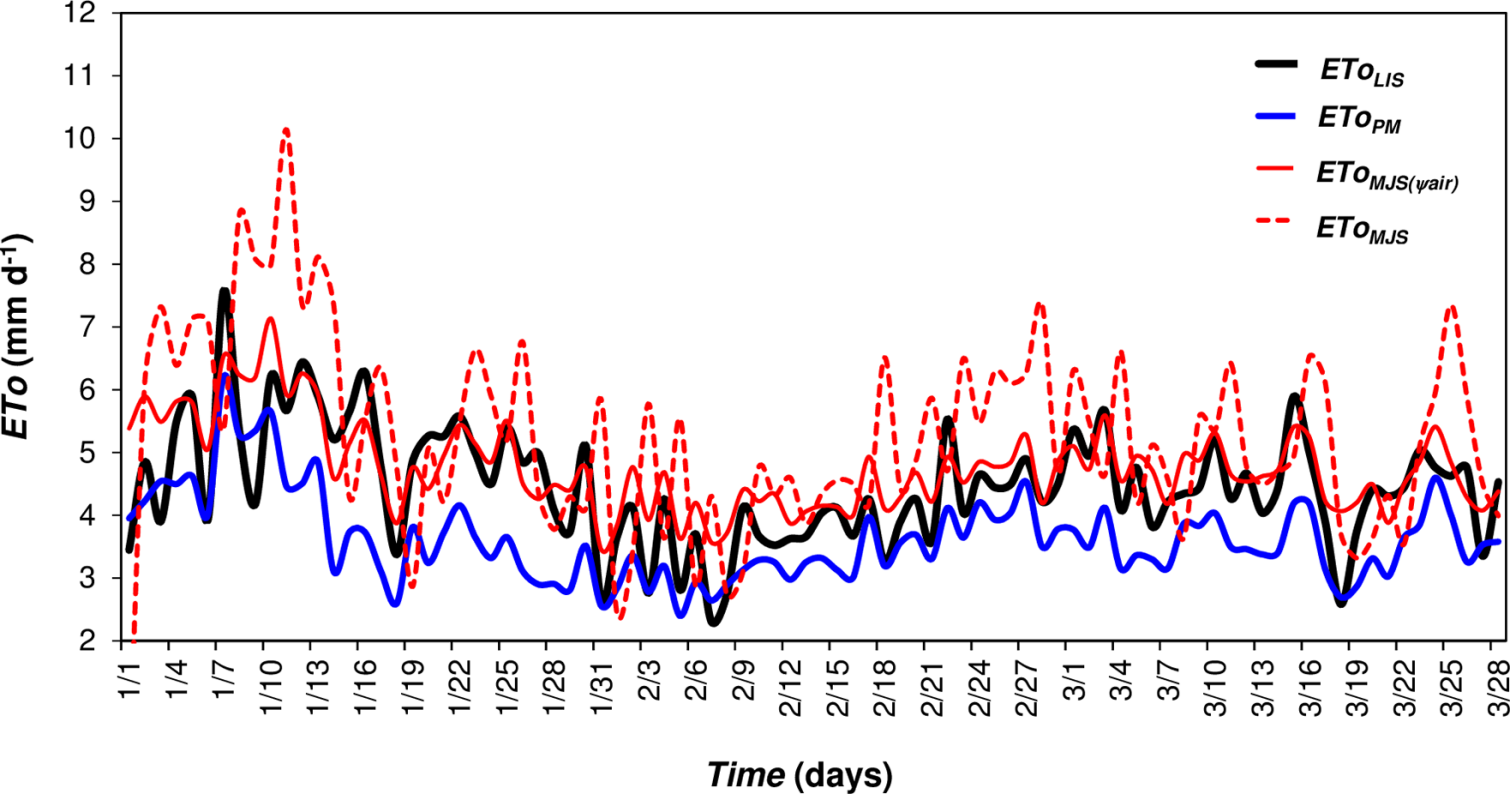
Daily *ETo*_*LIS*_, *ETo*_*PM*_, *ETo*_*MJS(Ψair)*_ and *ETo*_*MJS*_ for the semi-arid climate type *Bsh*, between 1997 and 1998 used as an in situ method of validation.

For the *in situ* validation, performed at the semiarid climate type *Bsh*, we identified a strong linear relationship (P<0.05) between *Ψ*_*air*_ and *ETo*_*LIS*_, which further supports the relationship identified between *ETo* and *Ψ*_*air*_ across all climatic zones (S1 Fig). We also identified strong agreement in the *ETo*_*MJS*(*Ψair*)_ and *ETo*_*MJS*_ estimated with respect to their monthly coefficients "*a*" and "*b*" (Fig 3 and Table 5). The highest error of adjustment obtained for *ETo*_*MJS*_ (MAE = 0.65 mm d^–1^) resulted in only 10% maximum overestimation of *ETo*_*LIS*_ at the reference site.

**Table 5:** Performance of *ETo*_*MJS(Ψair)*_ and *ETo*_*MJS*_ relative to measured *ETo*_*LIS*_ for the semi-arid climate type *Bsh* in an annual basis, between 1997 and 1998.

Adjustment	R	“d” Index	MAE	MR
	(dimensionless)		(mm d^–1^)	(dimensionless)
*ETo*_*MJS*(*Ψair*)_ *vs ETo*_*LIS*_	0.76	0.80	0.38	1.06
*ETo*_*MJS*_ *vs ETo*_*LIS*_	0.75	0.75	0.65	1.10

## Discussion

Our results demonstrate that extraterrestrial radiation and atmospheric water potential can be used to reliably estimate *ETo* in tropical and subtropical regions. The influence of other climatic variables needed for the standard *ETo*_*PM*_ calculation, such as *Rs* and *u*_*2*_, was indirectly but sufficiently accounted for in the analysis of radiation and atmospheric water potential, as evidenced by the strong agreement identified between *ETo*_*PM*_ and *ETo*_*MJS*_ estimates. Previous studies have shown similar results in cold and wet climates [54–55]; however, in our analysis of subtropical climates, the response of *ETo* to changes in *Ψ*_*air*_ indicates lower sensitivity relative to that observed in warmer and drier climates. In colder and wetter climatic zones, where atmospheric water demand is low (Table 4), *ETo* estimates were most strongly associated with *Rs* and *u*_*2*_ [72].

In tropical and semi-arid climates 1 MPa of variation in *Ψ*_*air*_ led to strong (up to 0.0861 mm d^–1^) *ETo*_*PM*_ responses. Similarly, we observed high sensitivity of the *ETo*_*MJS(Ψair)*_ method in response to air temperature and *RH*, which are the key variables controlling *Ψ*_*air*_ and used in climate classification systems [65]. This result was also confirmed in the analysis of “*a*” and “*b*” coefficients in warm climates (S1 Fig). Thus, the new simplified methods showed its best performances in warm and dry climatic zones (Fig 1 and S2 Fig)–with smaller errors of adjustment (R = 0.81 to 0.91; MAE: 0.34 to 0.58 mm d^–1^; and, “*d*” index = 0.88 to 0.95) than observed for subtropical climates (R = 0.48 to 0.71; MAE: 0.7 to 1.07 mm d^–1^; and, “*d*” index = 0.57 to 0.77). Considering the data spread of new and standard *ETo* estimates (relative to the 1:1 line in S2 Fig) our simplified method underestimated the *ETo*_*PM*_ by 1% in the semi-arid climate and overestimated the *ETo*_*PM*_ by up to 10% in the tropical climates and up to 16% in the subtropical climates (Table 4). Despite the high *Rs* observed in the semi-arid climate, the influence of *VPD* on *ETo*_*PM*_ was the most important, which is expected given the predominant warm and dry conditions [73–75], which led to strong agreement between *ETo*_*MJS(Ψair)*_ and *ETo*_*PM*_ at daily to seasonal scales.

In a previous study, the performance of 12 alternative *ETo* methods evaluated in 28 locations in central-western Brazil [46], the agreement (“*d*” index) of *ETo* obtained based on solar radiation (relative to the standard *ETo*_*PM*_) ranged from 0.32 to 0.91. In contrast, the agreement obtained here with the proposed *ETo*_*MJS*_ method ranged from 0.92 to 0.95 across the same climatic regions. Moreover, the *ETo*_*MJS*_ agreement index was higher (“*d*” index = 0.92 to 0.95) than observed with other alternative methods (“*d*” index = 0.50 to 0.82) [47]. Therefore, the use of solar radiation in the alternative methods significantly improved *ETo* estimates. Notably, we also observed higher associations between *ETo*_*MJS*_ and *ETo*_*PM*_ (R = 0.84 to 0.89) than reported in previous studies (R = 0.76 to 0.83) in subtropical humid climate of southern Brazil [41, 43]. In general, compared to the method based on atmospheric water potential (*ETo*_*MJS(Ψair)*_), the *ETo*_*MJS*_ method was more sensitive to seasonal and regional climate heterogeneity.

Our validation results showed the higher association and agreement between *ETo*_*MJS(Ψair)*_ vs *ETo*_*LIS*_ (R = 0.76 and “*d*” index = 0.80) and *ETo*_*MJS*_ vs *ETo*_*LIS*_ (R = 0.75 and “*d*” index = 0.75), using the coefficients “*a*” and “*b*” described above. Even the highest error of adjustment obtained for *ETo*_*MJS*_ (MAE = 0.65 mm d^–1^) resulted in low overestimations based on direct *ETo*_*LIS*_ measurements (<10%; Table 5). Since *VPD* is thought to has great influence on *ETo* in dry and warm conditions [36], the best performance of *ETo*_*MJS(Ψair)*_ is consistent with expected responses in semi-arid regions. As hypothesized, for such dry and warm climates, the best performance of *ETo*_*MJS(Ψair)*_ shows that *Ψ*_*air*_ and monthly average coefficients "*a*" and "*b*" grouped into climate subgroups, can be used to estimate *ETo* more confidently than in previous simplified models.

Comparing the findings in this study with previous tests of alternative *ETo* methods in other climatic regions, our *ETo*_*MJS(Ψair)*_ method also resulted in higher association and agreement with the standard *ETo*_*PM*_. For example, in tropical climate with dry winters (*Aw*) we obtained stronger agreement than previous simplifications based on either *RH* and air temperature (R = 0.49 to 0.83; “*d*” index = 0.49 to 0.69) [76] or based on solar radiation alone (R = 0.83, “*d*” index = 0.75) [40]. In addition, the association between *ETo*_*PM*_ and our alternative *ETo* estimates was stronger than those previously performed using *RH* and air temperature in other parts of the world [14, 73–75]. Methods based on solar radiation have been reported as a good alternative to the Penman-Monteith method either in humid [33, 51, 54, 77–79] and semi-arid climates [74, 80]. As it stands, these models present some difficulty of measurement [1, 5] due to scarce direct measurements and resulting large errors [31–32]. In our analysis, however, we show that even the sole use of *Ψ*_*air*_ in the alternative *ETo*_*MJS*(*Ψair*)_ method is sufficiently robust to estimate *ETo* in tropical and semi-arid climates. Moreover, the use of the solar radiation (*Ra*) in our method has proven to further improve *ETo* estimates, regardless of across all climatic zones studied here. Finally, the monthly average coefficients for climate subgroups improved the estimated *ETo*_*MJS*_, such that the results indicate the possibility of using of the monthly average “*a*” and “*b*” coefficients to expand the geography of estimates and assess the potential of land-to-air water losses across different climatic zones (Fig 2C and 2D).

Taking into account the well-known limitations of the existing alternative methods [81], such as applicability in regions of strong seasonality of across regions of that encompass multiple climates [10, 12, 81], another important improvement of the proposed method is its sensitivity to spatial variability of climate conditions. The use of the *Ψ*_*air*_–based estimates allows for investigating the spatial variability of *ETo*, which necessary to both conserve limited water resources as well as maintain food and energy production under changing climates [82–84].

## Conclusions

In this study, we present a new model to estimate reference evapotranspiration in tropical and subtropical regions, where the climatic information needed for the standard *ETo* calculation is scarce or absent. We describe how geographical and seasonal variability in evapotranspiration can be accurately predicted based on radiation and atmospheric water potential estimates. The new simplified method is particularly robust in tropical and semi-arid climates, but can also be applied in subtropical and wet climates. In all cases, the new method has significant benefits with respect to accuracy and spatiotemporal scale of application relative to previous models. Continued measurements of air temperature and relative humidity (needed for *Ψ*_*air*_ modeling) across different land uses will improve the accuracy of land-to-air water flux estimates in future studies.

## Supporting information

S1 Fig Daily reference evapotranspiration estimated by Penman-Monteith method as a response of atmospheric water potential (*Ψ*_*air*_), between 2004 and 2011, for the climate types: (a) Af; (b) Am; (c) As; (d) Aw; (e) Bsh; (f) Cfa; (g) Cfb; (h) Cwa; and, (i) Cwb.
(TIF)

S2 Fig Daily reference evapotranspiration estimated by Penman-Monteith method as a response of *ETo*_*MJS(*_*Ψ*_*air)*_, between 2012 and 2014, for the climate types: (a) Af; (b) Am; (c) As; (d) Aw; (e) Bsh; (f) Cfa; (g) Cfb; (h) Cwa; and, (i) Cwb.
(TIF)

S3 Fig Daily reference evapotranspiration estimated by Penman-Monteith method as a response of equivalent water evaporation (*E*_*e*_), between 2004 and 2011, for the climate types: (a) Af; (b) Am; (c) As; (d) Aw; (e) Bsh; (f) Cfa; (g) Cfb; (h) Cwa; and, (i) Cwb.
(TIF)

S4 Fig Daily reference evapotranspiration estimated by Penman-Monteith method as a response of *ETo*_*MJS*_ alternative method, between 2012 and 2014, for the climate types: (a) Af; (b) Am; (c) As; (d) Aw; (e) Bsh; (f) Cfa; (g) Cfb; (h) Cwa; and, (i) Cwb.
(TIF)
