# Supplementary figures and images for: Expanding the geography of evapotranspiration: An improved method to quantify land-to-air water fluxes in tropical and subtropical regions
Source: PLoS One. 2017 Jun 28;12(6):e0180055. doi: 10.1371/journal.pone.0180055 (PMC5489199; doi:10.1371/journal.pone.0180055)

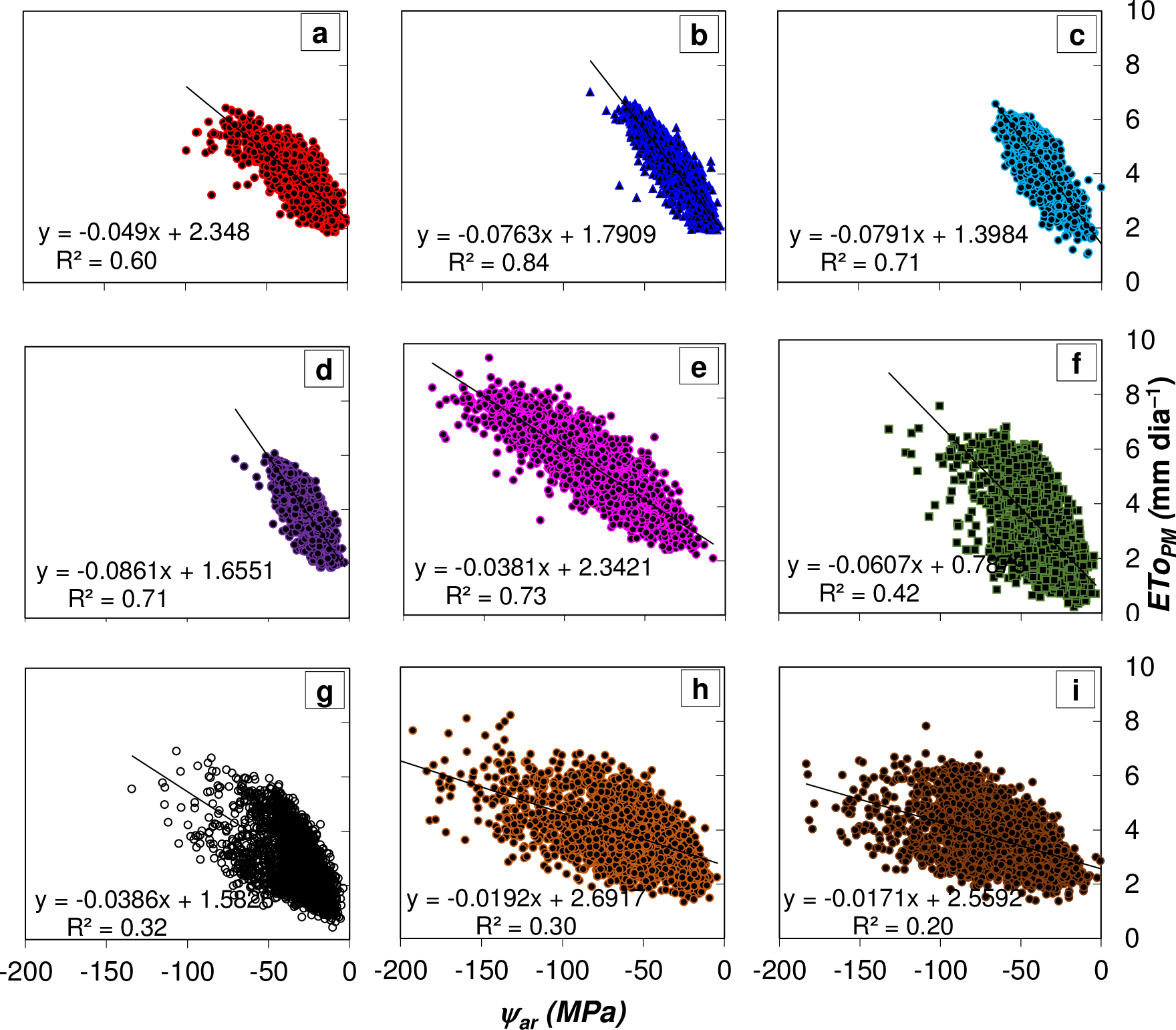

Supplement: S1 Fig — Daily reference evapotranspiration estimated by Penman-Monteith method as a response of atmospheric water potential (Ψair), between 2004 and 2011, for the climate types: (a) Af; (b) Am; (c) As; (d) Aw; (e) Bsh; (f) Cfa; (g) Cfb; (h) Cwa; and, (i) Cwb. (TIF) [file pone.0180055.s001.tif]

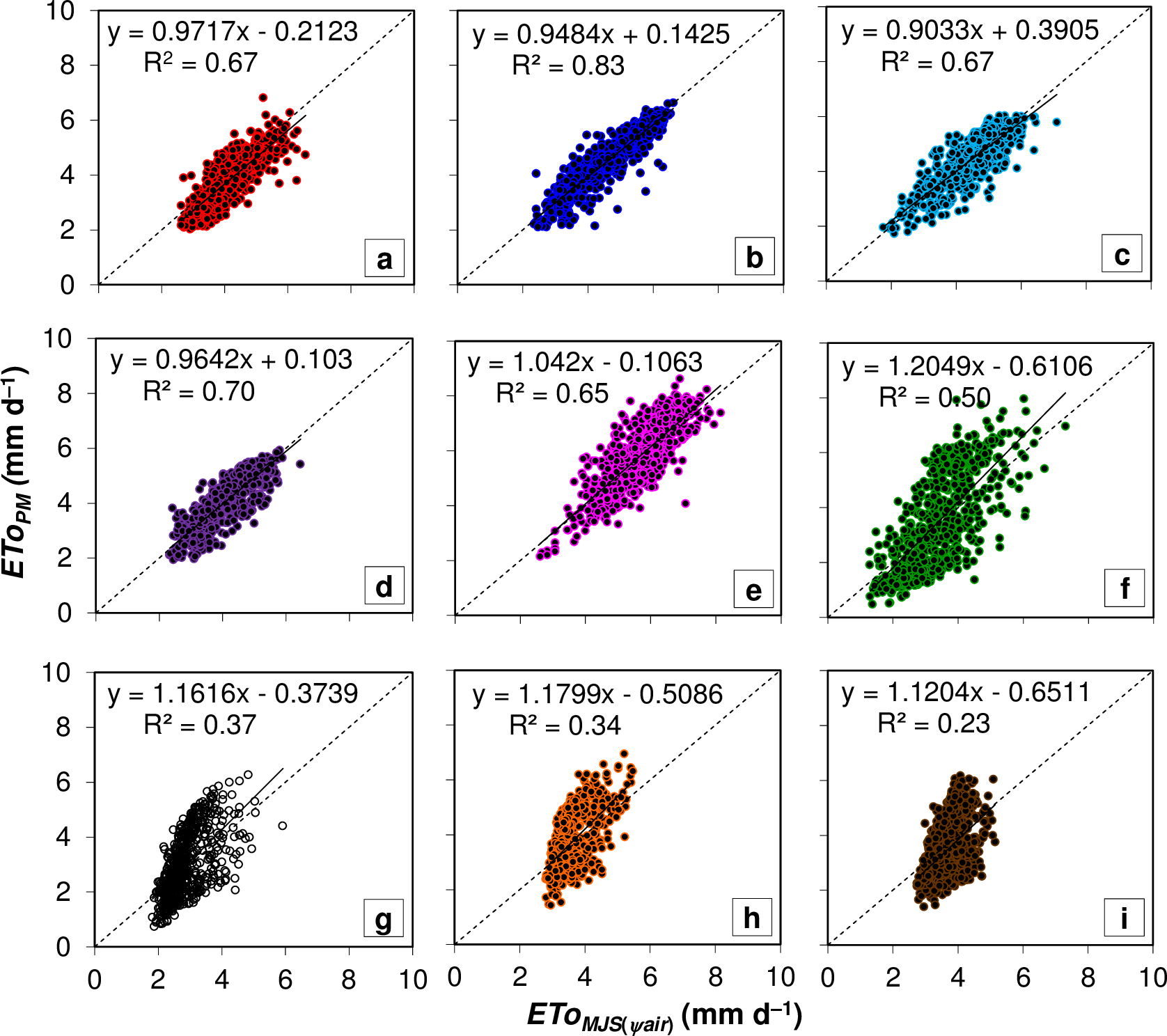

Supplement: S2 Fig — Daily reference evapotranspiration estimated by Penman-Monteith method as a response of EToMJS(Ψair), between 2012 and 2014, for the climate types: (a) Af; (b) Am; (c) As; (d) Aw; (e) Bsh; (f) Cfa; (g) Cfb; (h) Cwa; and, (i) Cwb. (TIF) [file pone.0180055.s002.tif]

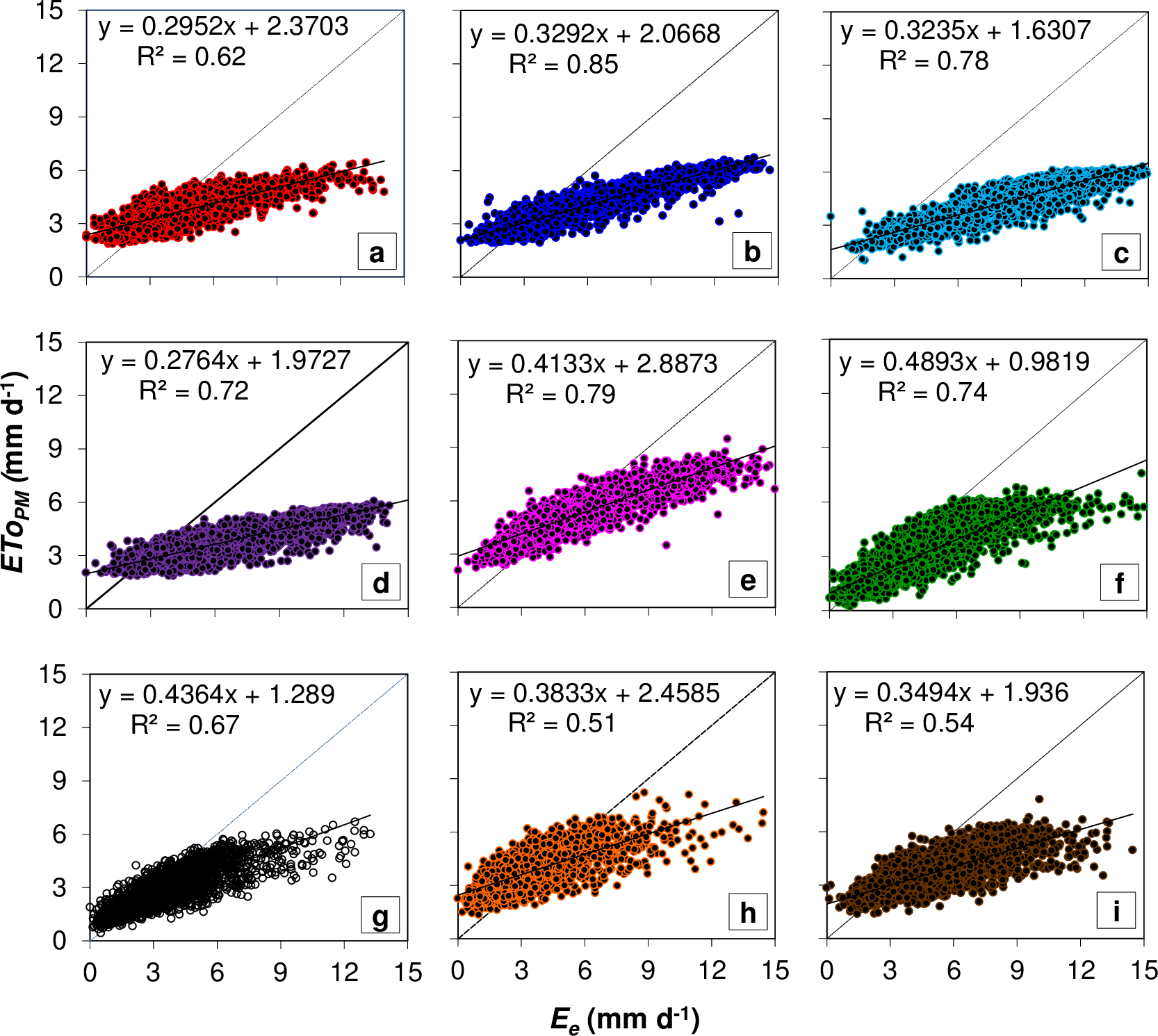

Supplement: S3 Fig — Daily reference evapotranspiration estimated by Penman-Monteith method as a response of equivalent water evaporation (Ee), between 2004 and 2011, for the climate types: (a) Af; (b) Am; (c) As; (d) Aw; (e) Bsh; (f) Cfa; (g) Cfb; (h) Cwa; and, (i) Cwb. (TIF) [file pone.0180055.s003.tif]

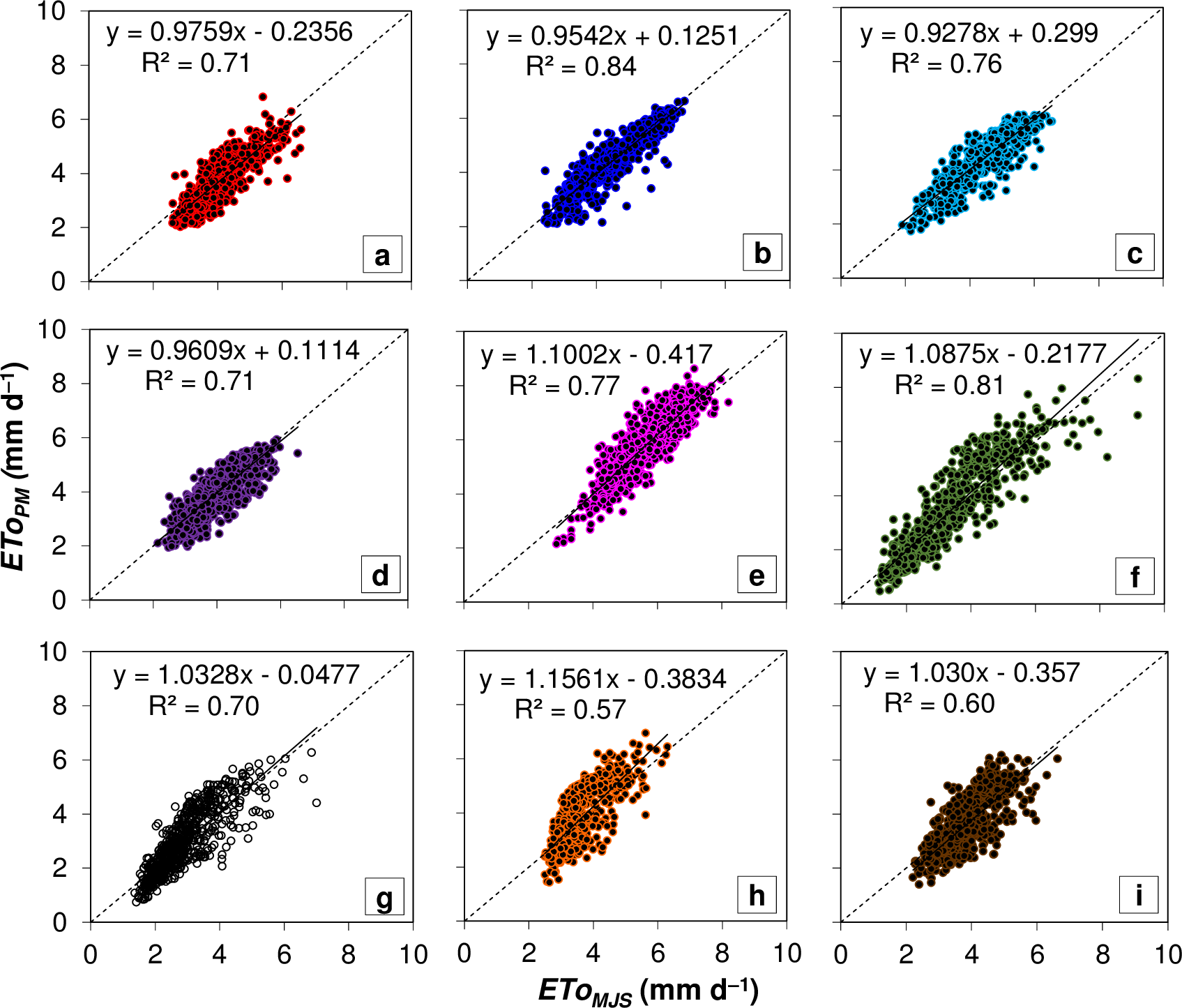

Supplement: S4 Fig — Daily reference evapotranspiration estimated by Penman-Monteith method as a response of EToMJS alternative method, between 2012 and 2014, for the climate types: (a) Af; (b) Am; (c) As; (d) Aw; (e) Bsh; (f) Cfa; (g) Cfb; (h) Cwa; and, (i) Cwb. (TIF) [file pone.0180055.s004.tif]
